# Vascular endothelial growth factor 165 inhibits pro-fibrotic differentiation of stromal cells via the DLL4/Notch4/smad7 pathway

**DOI:** 10.1038/s41419-019-1928-z

**Published:** 2019-09-12

**Authors:** Haining Lv, Ziqing Nan, Peipei Jiang, Zhiyin Wang, Minmin Song, Hailin Ding, Dan Liu, Guangfeng Zhao, Yaowu Zheng, Yali Hu

**Affiliations:** 1 Department of Obstetrics and Gynecology, Nanjing Drum Tower Hospital, Peking Union Medical College, Chinese Academy of Medical Science, Graduate School of Peking Union Medical College, 210008 Nanjing, China; 2 Department of Obstetrics and Gynecology, The Affiliated Drum Tower Hospital of Nanjing University Medical School, 210008 Nanjing, China; 3 Department of Obstetrics and Gynecology, Drum Tower Clinical Medical College, Nanjing Medical University, 210000 Nanjing, China; 4 Transgenic Research Center, School of Life Sciences, Northeast Normal University, 130000 Changchun, China; 5 Key Laboratory of Molecular Epigenetics of Ministry of Education, Northeast Normal University, 130000 Changchun, China

**Keywords:** Cell biology, Cell biology, Infertility, Infertility

## Abstract

Endometrial fibrosis is the main pathological feature of Asherman’s syndrome (AS), which is the leading cause of uterine infertility. Much is known about the expression of VEGF165 in luminal/glandular epithelial cells and stromal cells of the endometrium in normal menstrual cycles; however, less is known about the role and mechanism of VEGF165 in endometrial fibrosis. Herein, we report that VEGF165 is a key regulator in endometrial stromal cells to inhibit α-SMA and collagen 1 expression. Compared to human control subjects, patients with AS exhibited decreased VEGF165 expression in the endometrium along with increased fibrotic marker expression and collagen production. A fibrotic phenotype was shown in both mice with conditional VEGF reduction and VEGF165-deleted endometrial stromal cells. Exogenous VEGF165 could suppress TGFβ1-induced α-SMA and collagen 1 expression in human primary endometrial stromal cells. However, this beneficial effect was hindered when the expression of smad7 or Notch4 was inhibited or when Notch signaling was blocked, suggesting that smad7 and Notch4 are essential downstream molecules for VEGFA functioning. Overall, our results uncover a clinical targeting strategy for VEGF165 to inhibit pro-fibrotic differentiation of stromal cells by inducing DLL4/Notch4/smad7, which paves the way for AS treatment.

## Introduction

Asherman’s syndrome (AS), characterized by severe endometrial damage due to curettage, endometritis, or other unknown etiologies^1^, is a major cause of female secondary infertility^2^. Endometrial fibrosis, the primary pathological change in AS, is a process of abnormal tissue remodeling manifesting as aberrant collagen deposition and endometrial scarring/adhesion, which is related to the differentiation of endometrial stromal cells into myofibroblasts^3,4^.

Numerous factors produced by endometrial cells participate in the restoration of homeostasis during endometrial remodeling and regeneration^5^. Vascular endothelial growth factor A (VEGFA), whose predominant isoform is VEGF165, is one of the factors that is significantly upregulated in both the epithelium and stroma during postmenstrual repair and the early proliferative phase in the endometrium. As is well known, VEGFA is essential for rapid bursts of angiogenesis and regulates the reepithelization of the endometrium by participating in crosstalk between the epithelium and stroma^6,7^. Upregulation of VEGFA in the endometrium is usually an important indicator for evaluating therapeutic effects on endometrial injury^8,9^. Although it has been reported that impaired expression of VEGFA in hepatic, pulmonary or renal fibrosis further aggravates fibrotic phenotypes and poor prognoses^10–13^, the functions and mechanisms of VEGFA in endometrial fibrosis remain unclear.

Transforming growth factor β (TGFβ) signaling is a crucial target in antifibrosis therapy for the endometrium^4,14,15^. Smad7 is the central checkpoint and effective feedback regulator of TGFβ signaling^16^. Many studies on other organs have reported that blockade of TGFβ signaling through promotion of smad7 expression is a promising antifibrotic strategy^17^ that has a similar function to VEGF165 treatment^12^. However, whether and how VEGF165 exerts its antifibrotic function by regulating smad7 needs to be further explored.

In this study, we first investigated whether the expression of VEGF165 was significantly decreased in the endometrium in AS patients. In light of our observations, we hypothesized that impaired expression of VEGF165 contributes to endometrial fibrosis. The driving role of decreased VEGF165 in endometrial fibrosis was confirmed with cell experiments in vitro and with double transgenic (VEGF^tetO/tetO^/β-actin-tetR-Krab) mice, whose VEGFA expression could be effectively repressed and completely reversed by doxycycline (Dox)^18^. Mechanistic studies showed that endometrial fibrosis can be antagonized by VEGF165 through activation of the DLL4/Notch4/smad7 signaling pathway.

## Materials and methods

### Human endometrium biopsy

All human samples and procedures involved in this study were approved by the Ethics Committee of the Nanjing Drum Tower Hospital (No. 2012022).

One hundred patients who received services at the Infertility Consulting Clinic at the Nanjing Drum Tower Hospital were enrolled in this study, comprising 50 patients with AS aged 20–40 years old who were diagnosed by hysteroscopy and classified according to the American Fertility Society classification scoring method (The American Fertility Society, 1988) and 50 controls. Physical examinations were conducted to exclude women with tuberculosis. The clinical information for the patients is summarized in Table 1. All participants gave written informed consent after fully understanding the possibilities of procedural risks and failure.

**Table 1:** Patient information

	Control (*n* = 50)	AS (*n* = 50)	*P* value
Age (year)	30.7 ± 3.3	32.1 ± 3.9	>0.05
Duration of infertility (year)	3.6 ± 1.6	5.5 ± 1.6	<0.05
Indication	Chronic salpingitis 13/50 Hydrosalpinx 37/50	Intrauterine adhesion	
D&C (*n*)	2.5 ± 1.0	3.5 ± 1.4	<0.05
Times of hysteroscopy surgery	0	2.1 ± 1.0	<0.05
Endometrial thickness (mm)	7.8 **±** 0.7	5.1 ± 1.0	<0.05
Percentage of scar (%)	0	83.1 **±** 10.1	<0.05
AFS score	0	9.2 ± 1.4	<0.05

*AS* Asherman’s syndrome, *AFS* American Fertility Society, *D&C* dilatation and curettage

Anonymized human endometrial biopsies donated by the patients were obtained at the late proliferative phase of the menstrual cycle, which was determined according to the diameters of the follicles by ultrasound and the levels of progesterone in the blood. Individuals with 15–17 mm follicles and low progesterone levels were defined as being in the late proliferative phase^19^. In addition, the thickness of the endometrium was measured by ultrasound. If a patient was diagnosed with the moderate/severe type of AS under hysteroscopic evaluation, endometrial biopsies were obtained with biopsy instruments (Jinzhong, 15CrHBH040) from the anterior and posterior uterine walls, the fundus and the area of adhesion. If no sign of visible endometrial lesions under hysteroscopy were found and meanwhile the thickness of the endometrium was more than 7 mm, the patient was included in the control group, and endometrial biopsies were obtained from the anterior and posterior uterine walls and the fundus. The samples of each patient were divided into three portions: 1/4 was used for RNA isolation, 1/4 was used for protein extraction, and the remaining 1/2 was used for immunohistochemistry analysis.

### Animal housing and breeding

All animal experiments were carried out in accordance with the guidelines of the Experimental Animals Management Committee (Jiangsu Province, China) and were approved by the Ethics Review Board for Animal Studies of Nanjing Drum Tower Hospital.

Double transgenic (VEGF^tetO/tetO^/β-actin-tetR-Krab) mice were generated as described previously^18,20,21^. Briefly, four copies of tet operator (tetO) sequences were inserted into the promoter region of VEGFA by gene targeting. Transgenic mice with universal expression of tetR-Krab fusion protein were generated by nuclear DNA injection. Crossing the two lines yielded mice whose expression of VEGFA was controlled by the tetracycline analog Dox (Sangon Biotech, China). For such mice, when Dox is administered in food (Dox+), the tetR-Krab fusion protein binds to Dox and is removed from the VEGFA promoter, which causes the expression of VEGFA to return to normal following Mendelian inheritance. When Dox is removed (Dox−), the tetR-Krab fusion protein binds to the VEGFA promoter region and the target gene; thus, VEGFA is repressed^20^. The repression is reversible when the animal is switched back to Dox-containing food. For this experiment, the double transgenic mice ceased to be fed Dox-containing chow at weaning age and were switched to regular chow for at least 6 weeks before analysis. The control group was kept on Dox-containing chow until the time of assay^18^.

### Genotyping

Mouse tail samples approximately 0.5 cm in length were clipped from the transgenic mice and placed into EP tubes with 150 μL of 50 nM NaOH. After digestion in a 98 °C metal bath for 30 min, 1 M Tris-HCl (pH 8.0) was placed into the EP tubes, which were then vibrated and centrifuged. Then, DNA was obtained from the supernatant. The following pairs of primers were used for PCR: VEGFA, CGGCAGCGGAGCTCTGTCGC (forward) and AGCTCTTGATACCTCTTTCGT (reverse); tetR, CAGCGCATTAGTGCTGCTTA (forward) and TAGCGACTTGATGCTCTTGATC (reverse).

### Cell isolation, culture, and drug treatment

Endometrial tissues at the late proliferative phase from patients who underwent a hysterectomy were digested with a mixture of collagenase type I (Sigma, USA), DNase (Roche, Switzerland), and hyaluronidase (Sigma, USA) and size-fractionated with a 40-μm cell strainer (BD Biosciences, USA) to separate the fragments of the endometrial stromal cells from the glands. The endometrial stromal cells were inoculated into low-glucose Dulbecco’s modified Eagle's medium/Nutrient Mixture F-12 (DMEM/F12; Wisent Inc., Canada) containing 10% fetal bovine serum (FBS; Gibco, USA), 100 U/mL penicillin, and 100 μg/mL streptomycin (Wisent Inc., Canada) and cultured at 37 °C with 5% CO_2_ and saturated humidity. Cells from passages 2–4 were used for all experiments. The medium was changed every 2 to 3 days according to the growth of the cells. For stimulation experiments, cells were incubated with 2% FBS-DMEM/F12 medium containing recombinant human TGFβ1 (Catalog #AF-100-21C; PeproTech, USA) and VEGF165 (Catalog #293-VE; R&D Systems, USA). Wnt/β-catenin, Hedgehog, and Notch signalings were inhibited in cells using KYA1797K (Catalog #HY-101090; MedChemExpress, USA), cyclopamine (Catalog #HY-17024; MedChemExpress, USA), and DAPT (Catalog #HY-13027; MedChemExpress, USA), respectively.

### RNA isolation and quantitative real-time PCR (qPCR)

Total RNA from the tissues or cultured cells was extracted with TRIzol Universal Reagent (Tiangen, China). One microgram of RNA was reverse-transcribed into cDNA using 5× All-In-One RT MasterMix (Applied Biological Materials Inc., Canada). Individual qPCR mixes were made according to the recommendations of the manufacturer of ChamQ SYBR® qPCR Master Mix (Without ROX) (Vazyme, China). Differences among the target gene expression levels were estimated by the ΔΔ*C*_t_ method, and target gene expression was normalized by GAPDH expression. The values are the mean ± SEM. The primers used in this study are listed in Supplementary Table S1.

### Gene silencing of VEGFA, smad7, Notch1, and Notch4 using siRNA

Cells at approximately 50% confluence were transfected using Lipofectamine 2000 according to the manufacturer’s guidelines (Invitrogen, USA). siRNA against VEGF165 (siVEGF), smad7 (siSmad7), Notch1 (siNotch1), and Notch4 (siNotch4) were designed, produced, and annealed by Guangzhou Ribobio Co., Ltd. (China). The sequence information is as follows:

siVEGF165: 5′-UCACCGCCUCGGCUUGUCATT-3′,

siSmad7: 5′-GGACGCTGTTGGTACACAA-3′,

siNotch1: 5′-GCACGCGGAUUAAUUUGCAdTdT-3′, and

siNotch4: 5′-GGCGGACGUCGCUCACCAAdTdT-3′.

### Western blot analysis

Tissues or cells were lysed in lysis buffer (Biosharp, China) supplemented with protease inhibitor cocktail and phosphatase inhibitor cocktail (MedChemExpress, USA) and clarified by centrifugation at 12,000 × *g* for 15 min. The protein concentrations were determined using a Pierce BCA protein assay kit (Thermo Scientific, USA). Protein samples (30 μg) were fractionated with SDS-PAGE, transferred to PVDF membranes (Bio-Rad, USA), and incubated in 5% nonfat milk (Bio-Rad, USA) at room temperature. The membranes were incubated with primary antibodies at 4 °C overnight and were then incubated with HRP-conjugated secondary antibodies at room temperature for 1 h. The signals were visualized with ECL solution (Bio-Rad, USA) and quantified by analysis of the integrated density normalized to the level of β-actin using ImageJ. The antibodies used are shown in Supplementary Table S2.

### Immunohistochemistry

Paraffin blocks were cut into slices at a thickness of 2 μm, which were collected on polylysine-coated glass slides. Endogenous peroxidase activity was blocked with 3% H_2_O_2_, and antigens were retrieved with universal antigen retrieval solution for immunohistochemistry (Typing, China). The slices were incubated with primary antibodies, which were diluted in antibody diluent reagent solution (Life Technologies, USA), at 4 °C overnight. The sections were incubated with HRP-conjugated secondary antibodies, exposed to DAB to visualize the antigen signals, and counterstained with hematoxylin. After the sections were sealed with a neutral resin, the positive reactants were viewed under a microscope (DM6 B, Leica, Germany). Immunohistochemical staining was quantified by mean optical density using Image-Pro Plus, and five random fields per sample were used for counting. The antibodies used in this study are listed in Supplementary Table S2.

### Immunofluorescence

Human endometrial stromal cells were fixed in 4% paraformaldehyde and then washed in phosphate-buffered saline (PBS). After permeabilization with 0.2% Triton X-100 in PBS (PBST), the cells were blocked in 1% bovine serum albumin/PBST. Primary antibody and secondary antibody incubations were performed for 60 min at room temperature in sequence. Nuclei were counterstained using 4′,6-diamidino-2-phenylindole (DAPI; Abcam). The staining was viewed using a microscope (DM6 B; Leica, Germany) and quantified by mean optical density using Image-Pro Plus. Five random fields per sample were used for counting. The antibodies used in this study are listed in Supplementary Table S2.

### Hydroxyproline content

To analyze collagen production, 10 mg of tissue or cells was homogenized in 100 μL of water, incubated in hydrochloric acid at 120 °C for 3 h, and then processed per the manufacturer protocols (Sigma Aldrich, USA). The absorbance at 560 nm was measured using a Multiskan GO microplate spectrophotometer (Thermo Scientific, USA). Hydroxyproline content was calculated using a standard curve.

### Statistics

Statistical analyses were performed using GraphPad Prism (Version 5.0, USA). An unpaired *t*-test was used with or without Welch’s correction depending on the variance of data. ANOVA with SNK-q’s post hoc test was used for multiple comparisons, and *P* < 0.05 was considered to indicate statistical significance.

## Results

### Decreased expression of VEGF165 is associated with endometrial fibrosis in AS patients

**Fig. 1:**
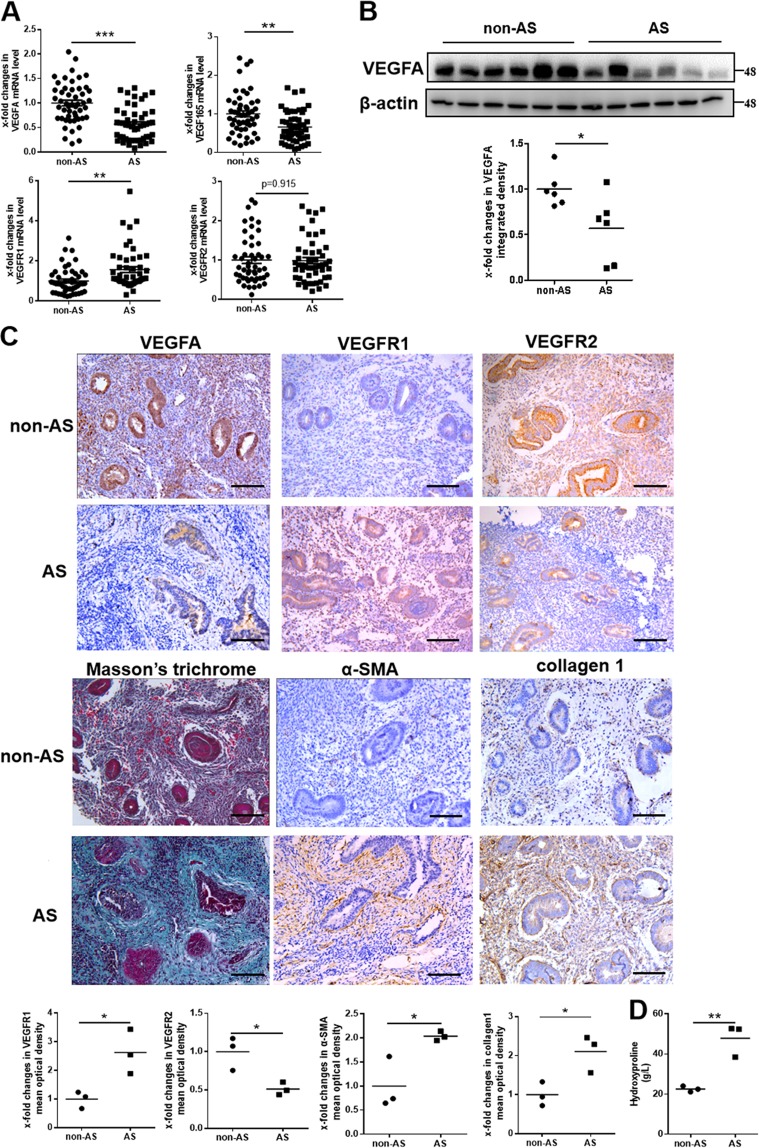
Endometrial fibrosis in AS patients is related to decreased expression of VEGFA. **a** The mRNA expression levels of VEGFA, VEGF165 and two receptors, VEGFR1 and VEGFR2, relative to the control (GAPDH) in endometria from human subjects with AS (*N* = 50) and without AS (non-AS; *N* = 50) were examined by qPCR. Each bar represents the mean ± SEM. ***P < 0.001, **P < 0.01. Unpaired *t*-test. Analysis of VEGF165 and VEGFR1 was performed using Welch’s correction. **b** The protein levels of VEGFA were significantly decreased in the endometria of AS patients (*N* = 6) compared to non-AS patients (*N* = 6). *P < 0.05. Unpaired *t*-test. **c** Paraffin-embedded human endometrial tissues were analyzed by immunohistochemistry for VEGFA, VEGFR1, VEGFR2, α-SMA, or collagen 1 and by Masson’s trichrome staining at the late proliferative phase. All images are magnified ×200. Scale bar = 100 μm. *N* = 3, *P < 0.05. Unpaired *t*-test. **d** Hydroxyproline content was used to confirm endometrial fibrosis. *N* = 3, **P < 0.01. Unpaired *t*-test. AS Asherman’s syndrome

The expression patterns of VEGF165 and its two receptors, VEGFR1 and VEGFR2, were analyzed in endometrial biopsies of AS patients and controls. The mRNA levels of VEGFA and VEGF165 in the endometria of patients with AS (*n* = 50) were significantly lower than those in matched non-AS individuals (*n* = 50), as determined by qPCR. Although the mRNA levels of VEGFR1 were increased in patients with AS, the levels of VEGFR2 were not different between patients with AS and controls (Fig. 1a). These changes were confirmed by western blot analysis (Fig. 1b) and immunohistochemistry (Fig. 1c, Supplementary Figs. S1–3). Upregulated expression of α-smooth muscle actin (α-SMA) and collagen 1 in the endometrial stroma and increased hydroxyproline content and Masson’s trichrome staining in the endometrium showed the presence of endometrial fibrosis in the patients with AS (Fig. 1c, d).

### VEGF164 repression aggravates endometrial fibrosis

**Fig. 2:**
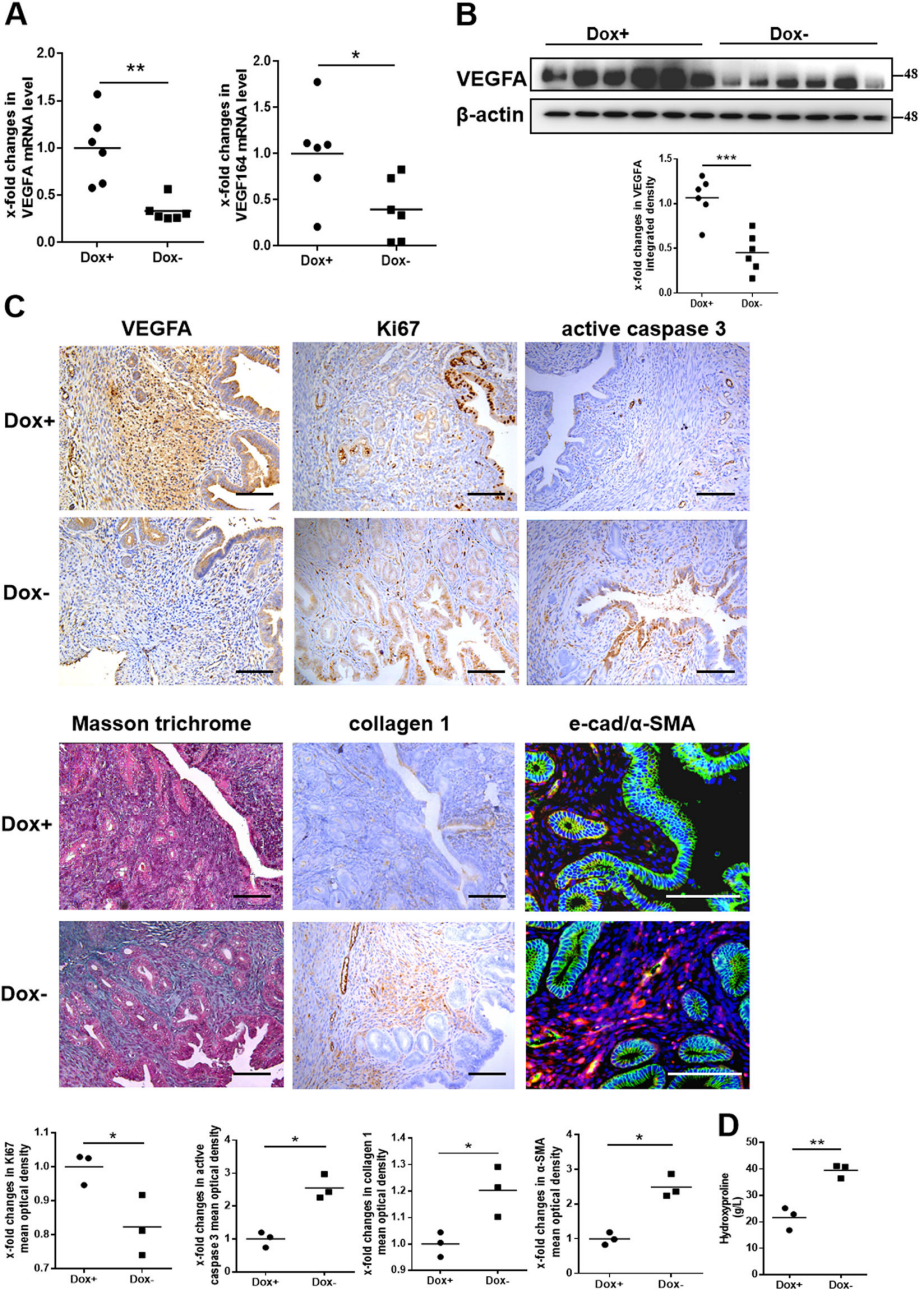
VEGFA repression induces endometrial fibrosis in mice. **a** The mRNA levels of VEGFA and VEGF164 in estrous uteri from Dox+ (*N* = 6) and Dox− mice (*N* = 6) were examined by qPCR. *P < 0.05, **P < 0.01. Unpaired *t*-test. **b** Uterine protein levels of VEGFA were significantly decreased in Dox− mice compared to Dox+ mice. *N* = 6, ***P < 0.001. Unpaired *t*-test. **c** Representative micrographs showing VEGFA, Ki67, active caspase 3, and collagen 1 levels by immunohistochemistry, e-cad (green), and α-SMA (red) levels by immunofluorescence, and Masson’s trichrome staining of murine uteri in estrus. Scale bar = 100 μm. *N* = 3, *P < 0.05. Unpaired *t*-test. **d** Hydroxyproline content was used to confirm uterine fibrosis. *N* = 3, **P < 0.01. Unpaired *t*-test

We then investigated the effects of reduced VEGFA on the endometrium using transgenic mice. Compared with those in Dox + mice (*n* = 6), the uterine mRNA and protein levels of VEGFA and its main isoform, VEGF164 (homologous to VEGF165 in humans), were significantly reduced in Dox− mice at estrus (*n* = 6), as determined by both qPCR and western blot analysis (Fig. 2a, b). Immunohistochemical staining revealed that the signal of decreased VEGFA expression was mainly located in the stroma (Fig. 2c, Supplementary Figs. S1 and 4). To explore the effect of the decreased VEGFA in the stroma of the murine uterus, we examined the expression of Ki67, active caspase 3, and markers of fibrosis (collagen 1 and α-SMA) by immunohistochemical staining. The results showed that Ki67 expression was decreased significantly in Dox− mice compared to Dox+ mice, while active caspase 3 expression was increased dramatically, which indicated that proliferation of endometrial cells was inhibited and apoptosis was increased in Dox− mice. Notably, decreased expression of VEGFA in the endometrial stroma could lead to upregulated expression of collagen 1 and α-SMA as well as increased hydroxyproline content and Masson’s trichrome staining in Dox− murine uteri, suggesting the existence of fibrosis in Dox− mice compared to Dox+ mice (Fig. 2c, d, Supplementary Figs. S1 and 4). From the above results, we concluded that repression of VEGFA expression in the stroma participated in murine endometrial fibrosis.

### Silencing VEGF165 in endometrial stromal cells induces the increased expression of α-SMA and collagen 1, which is associated with downregulation of smad7

**Fig. 3:**
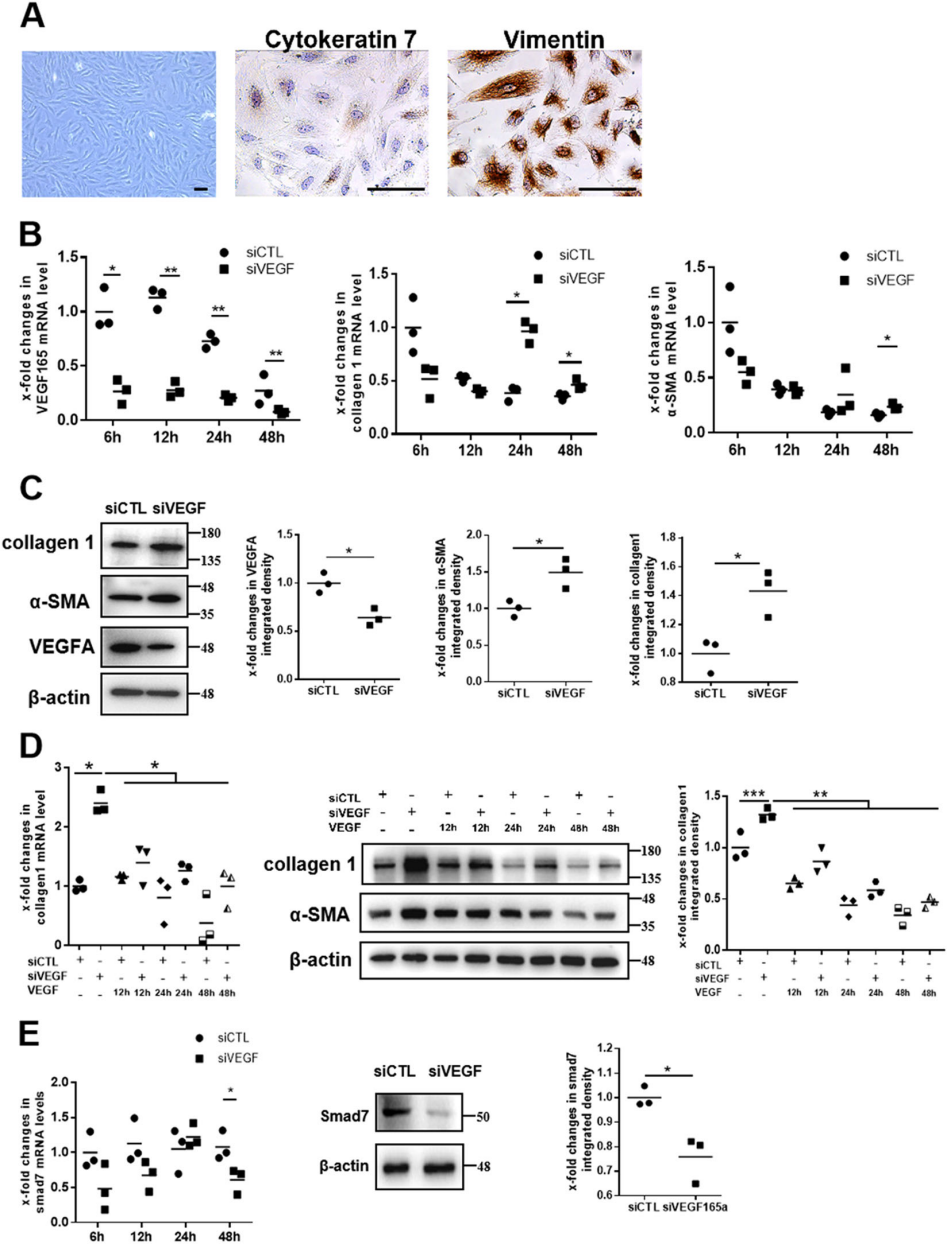
VEGF165 suppression leads to increased expression of α-SMA and collagen 1 and decreased expression of smad7 in endometrial stromal cells. **a** Human endometrial stromal cells were identified through morphological observation of the cells and through immunohistochemistry for cytokeratin 7 and vimentin. Scale bar = 100 μm. **b** The mRNA expression levels of VEGFA, collagen 1, and α-SMA in endometrial stromal cells were examined by qPCR at 6, 12, 24, and 48 h after transfection. *N* = 3, **P < 0.01, *P < 0.05. Unpaired *t*-test. c The protein levels of VEGFA, collagen 1, and α-SMA in endometrial stromal cells were examined by western blot analysis at 48 h after siVEGF165 transfection. *N* = 3, *P < 0.05. Unpaired *t*-test. **d** qPCR and western blot analysis confirmed that treatment with VEGF165 reversed the increased collagen 1 expression in endometrial stromal cells after VEGF165 suppression. *N* = 3, ***P < 0.001, **P < 0.01, *P < 0.05. Two-way ANOVA and SNK-q. **e** The mRNA expression of smad7 in human endometrial stromal cells was examined by qPCR at 6, 12, 24, and 48 h after transfection with siVEGF165. The protein levels of smad7 were examined by western blot analysis at 48 h after transfection with siVEGF165. *N* = 3, *P < 0.05. Unpaired *t*-test. siCTL siControl, siVEGF siVEGF165, VEGF VEGF165

The purity of human primary endometrial stromal cells was assessed using the difference in vimentin and cytokeratin 7 expression. The results of cell immunohistochemistry showed that the purity of the endometrial stromal cells was greater than 95%, indicating that the cells could be used for subsequent experiments (Fig. 3a). To investigate the effects of low levels of VEGF165 on endometrial stromal cells, VEGF165-specific small interfering RNA (siVEGF) was transfected into stromal cells. We confirmed that VEGF165 mRNA levels were significantly reduced at 6, 12, 24, and 48 h after transfection (Fig. 3b). As expected, collagen 1 mRNA levels were significantly increased in stromal cells transfected with siVEGF compared to those transfected with siControl (siCTL) at 24 and 48 h after transfection (Fig. 3b). α-SMA mRNA levels were dramatically upregulated at 48 h after transfection (Fig. 3b). The changes in VEGFA, α-SMA, and collagen 1 at 48 h after transfection were also confirmed by western blot analysis (Fig. 3c). The changed morphology of stromal cells between transfecting with siCTL and siVEGF was found and the cells transfected with siCTL manifested elongated shape, while cells transfected with siVEGF exhibited appearance of stress fibers (Supplementary Fig. S5). Notably, when the changed stromal cells were treated with 10 ng/mL VEGF165 for 12, 24, and 48 h, the cell phenotype could be changed to a near-normal phenotype (Fig. 3d).

Smad7, a negative feedback inhibitor of TGFβ/Smad canonical signaling, can compete with Smad2 and Smad3 for binding to activated TGFRI and thus reduce TGFβ/Smads signaling and inhibit the expression of fibrotic genes, such as collagen 1 and α-SMA^22^. The mRNA and protein levels of smad7 in cultured endometrial stromal cells decreased significantly after transfection with siVEGF for 48 h (Fig. 3e).

### VEGF165 reverses TGFβ1-stimulated α-SMA and collagen 1 expression in endometrial stromal cells

**Fig. 4:**
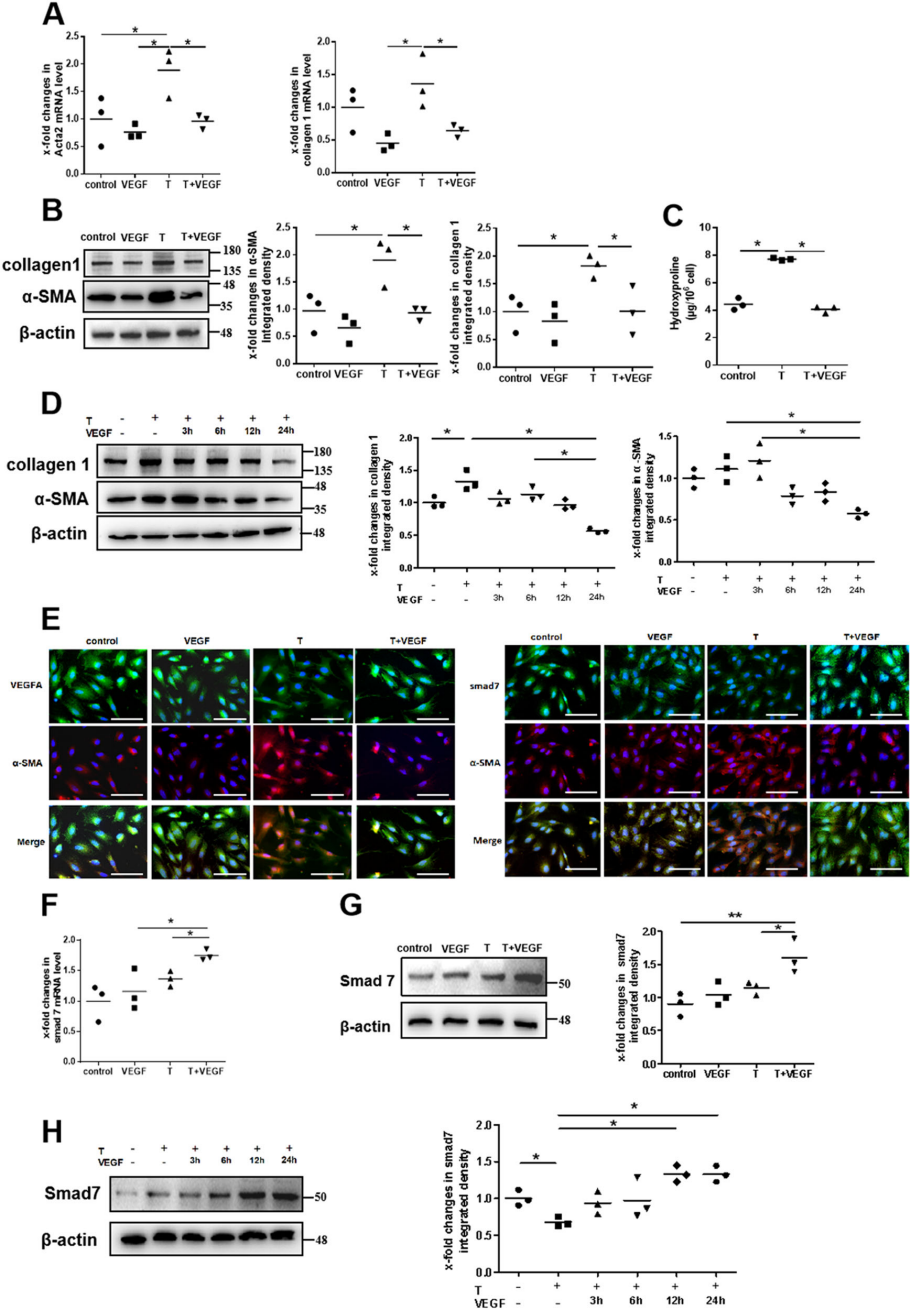
VEGF165 reverses TGFβ1-stimulated fibrotic gene expression and increased smad7 expression in endometrial stromal cells. Endometrial stromal cells (ESCs) were cultured in the presence of 10 ng/mL TGFβ1 for 48 h and/or cocultured with 10 ng/mL VEGF165 for another 24 h. ESCs were examined by qPCR (**a**) and western blot analysis (**b**) for expression of α-SMA and collagen 1. *N* = 3, *P < 0.05. One-way ANOVA and SNK-q. **c** Hydroxyproline content was used to confirm the differentiation of stromal cells into myofibroblasts. One-way ANOVA and SNK-q. **d** The protein levels of α-SMA and collagen 1 were examined by western blot analysis after ESCs were treated with 10 ng/mL TGFβ1 for 48 h and/or treated with 10 ng/mL VEGF165 for another 3, 6, 12, or 24 h. *N* = 3, *P < 0.05. Two-way ANOVA and SNK-q. **e** Representative imag**e**s of VEGFA, smad7, and α-SMA immunofluorescence staining in ESCs under different treatments. VEGFA, smad7: green; α-SMA: red; nuclei: blue. *N* = 3, scale bar = 100 μm. **f**, **g** ESCs were examined by qPCR and western blot analysis for expression of smad7. *N* = 3, **P < 0.01, *P < 0.05. One-way ANOVA and SNK-q. **h** The protein levels of smad7 were examined by western blot analysis after ESCs were treated with 10 ng/mL TGFβ1 for 48 h and/or treated with 10 ng/mL VEGF165 for another 3, 6, 12, or 24 h. *N* = 3, *P < 0.05. Two-way ANOVA and SNK-q. T TGFβ1, VEGF VEGF165

As is well known, TGFβ1 can promote fibroblasts to differentiate into myofibroblasts, causing fibrosis to occur. We applied TGFβ1-induced α-SMA and collagen 1 expression as a model to verify whether VEGF165 had antifibrotic effects. As the results showed, the mRNA expression levels of α-SMA and collagen 1 significantly increased after the cells were stimulated with TGFβ1 (10 ng/mL), while they significantly decreased after the cells were subsequently treated with VEGF165 (10 ng/mL) (Fig. 4a). These results were confirmed by western blot analysis (Fig. 4b). Hydroxyproline content analysis suggested that production of collagen was increased in TGFβ1-treated stromal cells and that VEGF165 reversed this change (Fig. 4c). In addition, VEGF165 could lead to reversal of the TGFβ1-induced elevations in collagen 1 and α-SMA levels in a time-dependent manner (Fig. 4d). Immunofluorescence for VEGFA and α-SMA expression demonstrated cytoplasmic localization and confirmed the changes (Fig. 4e), which indicated that VEGF165 could prevent the TGFβ1-induced α-SMA and collagen 1 expression.

### The protective effect of VEGF165 is impaired after the expression of smad7 is disrupted

To explore the mechanism by which VEGF165 reverses TGFβ1-stimulated α-SMA and collagen 1 expression, we examined the role of smad7 in this process. The results indicated that TGFβ1 could induce smad7 expression, which could not reduce the expression of collagen 1 (Supplementary Fig. S6). In contrast, VEGF165 not only promoted smad7 expression when used alone but also further strengthened the upregulation of smad7 expression induced by TGFβ1 and led to decreased expression of collagen 1 and α-SMA, which resulted in efficient antifibrotic effects in endometrial stromal cells (Fig. 4d–h, Supplementary Fig. S6). The pattern of increased expression of smad7 in stromal cells was confirmed by immunofluorescence, which suggested that smad7 was mainly located in the cytoplasm (Fig. 4e).

**Fig. 5:**
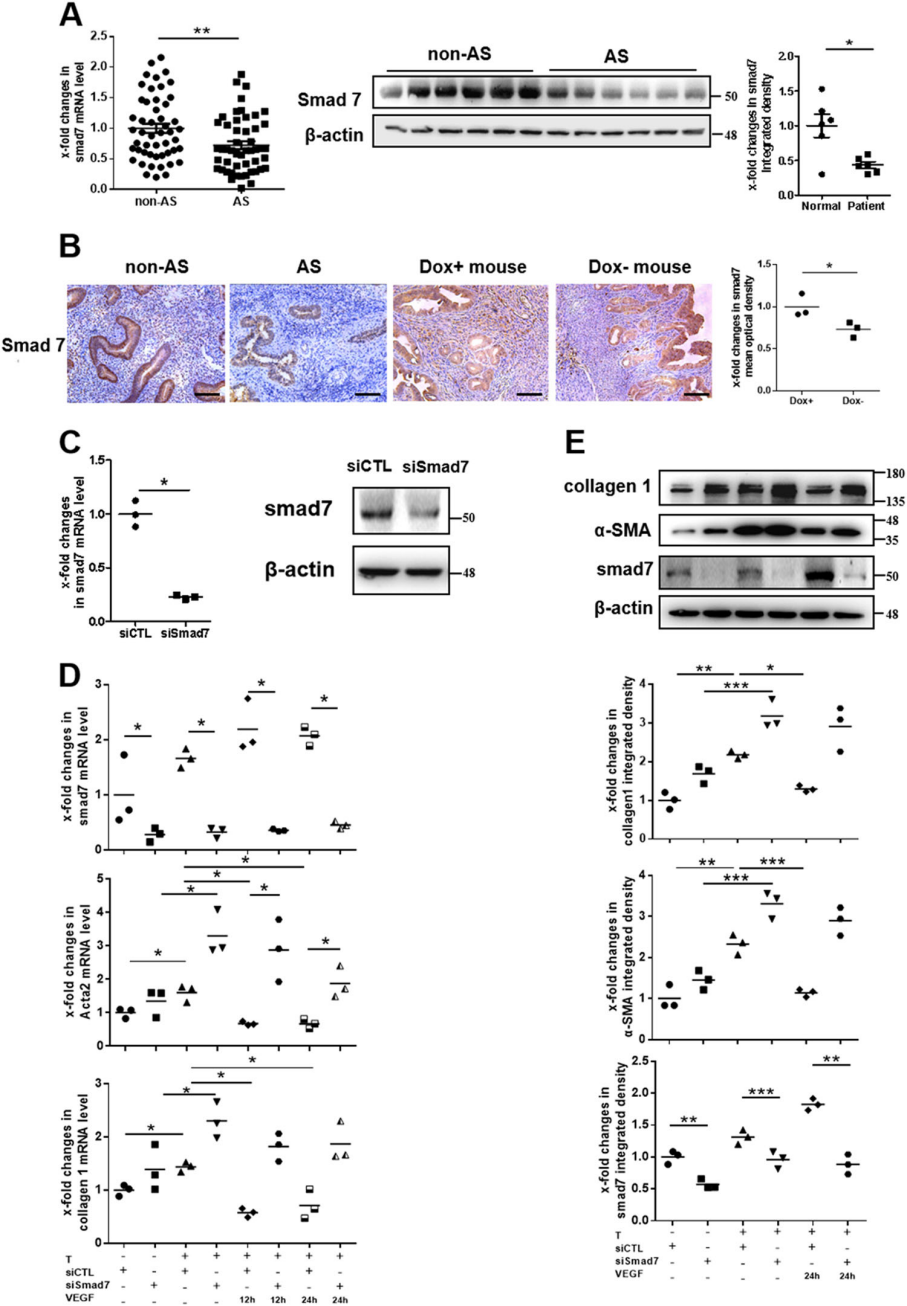
VEGF165 promotes smad7 expression to inhibit the differentiation of endometrial stromal cells into myofibroblasts. **a** The mRNA levels of smad7 in endometria from human subjects with AS (*N* = 50) and without AS (non-AS; *N* = 50) were examined by qPCR. The protein levels of smad7 as examined by western blot analysis were significantly decreased in the endometria of AS patients (*N* = 6) compared to non-AS patients (*N* = 6). Each bar represents the mean ± SEM. **P < 0.01, *P   <  0.05. Unpaired *t*-test. Analysis of the protein levels of smad7 was performed using Welch’s correction. **b** Representative micrographs showed smad7 levels in human endometria at the late proliferative phase and in murine uteri in estrus by immunohistochemistry. Scale bar = 100 μm, *N* = 3, *P < 0.05. Unpaired *t*-test. **c** The mRNA and protein levels of smad7 in human endometrial stromal cells were decreased significantly at 24 h after transfection with siSmad7. *N* = 3, *P < 0.05. Unpaired *t*-test. **d**, **e** Endometrial stromal cells stimulated with 10 ng/mL TGFβ1 for 48 h were transfected with siSmad7 for 24 h and/or cocultured with 10 ng/mL VEGF165 for another 12 or 24 h. The mRNA and protein levels of smad7, α-SMA, and collagen 1 were examined. *N* = 3, ***P < 0.001, **P < 0.01, *P < 0.05. Analysis of mRNA levels was performed using two-way ANOVA and SNK-q. Analysis of protein levels was performed using one-way ANOVA and SNK-q. AS: Asherman’s syndrome, T TGFβ1, VEGF VEGF165, siCTL siControl

The mRNA and protein levels of smad7 were generally downregulated in the endometria of AS patients (Fig. 5a). The patterns of the decreased expression of smad7 in AS endometria and Dox− murine uteri were confirmed by immunohistochemistry, which indicated that smad7 levels were mainly decreased in stroma cells (Fig. 5b, Supplementary Figs. S1, 2 and 4). To investigate the contribution of smad7 to the protective effect of VEGF165 against endometrial fibrosis, small interfering RNA-mediated knockdown of smad7 (with siSmad7) was employed in stromal cells. As expected, siSmad7 efficiently reduced smad7 expression in siSmad7-treated stromal cells compared to negative control cells, as shown by qPCR and western blot analysis (Fig. 5c). We next assessed the impact of siSmad7 on the expression of collagen 1 and α-SMA in endometrial stromal cells induced by TGFβ1 followed by VEGF165. The results showed that siSmad7 treatment blocked the VEGF165-mediated reversal of TGFβ1-induced elevations in collagen 1 and α-SMA compared to siControl treatment (Fig. 5d, e), indicating the crucial role of smad7 in the antifibrotic function of VEGF165.

### Notch signaling activation is essential for maintaining smad7 expression induced by VEGF165

**Fig. 6:**
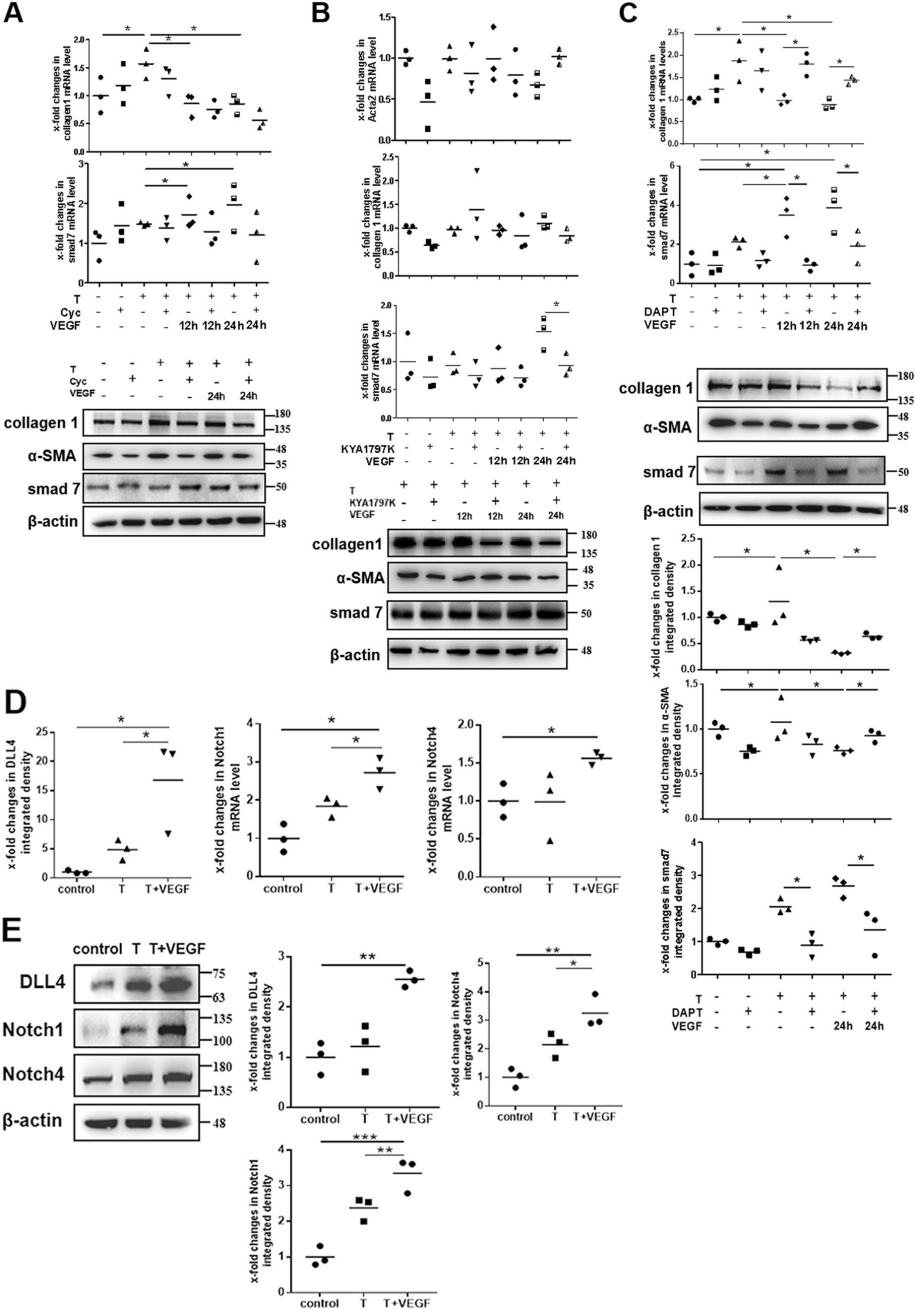
Notch signaling activation is crucial for VEGF165-mediated anti-fibrotic differentiation of stromal cells. **a** Endometrial stromal cells were stimulated with 10 ng/mL TGFβ1 for 48 h. Then these cells were treated with 10 μM Cyc for 24 h and/or cocultured with 10 ng/mL VEGF165 for another 12 or 24 h. The mRNA or protein levels of collagen 1, α-SMA and smad7 were examined. N=3, *P < 0.05. Analysis of mRNA levels was performed using two-way ANOVA and SNK-q. Analysis of protein levels was performed using one-way ANOVA and SNK-q. **b** Endometrial stromal cells were stimulated with 10 ng/mL TGFβ1 for 48 h. Then these cells were treated with 25 μM KYA1797K for 24 h and/or cocultured with 10 ng/mL VEGF165 for another 12 or 24 h. The mRNA or protein levels of collagen 1, α-SMA and smad7 were examined. N=3, *P < 0.05. Two-way ANOVA and SNK-q. **c** Endometrial stromal cells were stimulated with 10 ng/mL TGFβ1 for 48 h. Then these cells were treated with 30 μM DAPT for 24 h and/or cocultured with 10 ng/mL VEGF165 for another 12 or 24 h. The mRNA or protein levels of collagen 1, α-SMA and smad7 were examined. N=3, *P < 0.05. Analysis of mRNA levels was performed using two-way ANOVA and SNK-q. Analysis of protein levels was performed using one-way ANOVA and SNK-q. **d**-**e** Endometrial stromal cells stimulated with 10 ng/mL TGFβ1 for 48 h were treated with 10 ng/mL VEGF165 for another 24 h. The mRNA or protein levels of DLL4, Notch1 and Notch4 were examined. N=3, *P < 0.05. One-way ANOVA and SNK-q. T TGFβ1, VEGF VEGF165, Cyc cyclopamine

The Wnt/β-catenin, Hedgehog, and Notch signaling pathways are known to participate in maintaining cell development, differentiation, proliferation, and apoptosis^23,24^. The involvement of these signaling pathways in smad7 expression was evaluated with inhibitors, including KYA1797K (an inhibitor of Wnt/β-catenin signaling), cyclopamine (Cyc, an inhibitor of Hedgehog signaling), and DAPT (an inhibitor of Notch signaling). Dosage response experiments were first performed to confirm the effects of the inhibitors (Supplementary Fig. S7), and according to the results, we chose 10 μM Cyc, 25 μM KYA1797K, and 30 μM DAPT for the following experiments. As depicted in Fig. 6a–c, treatment with Cyc (10 μM) (Fig. 6a) and KYA1797K (25 μM) (Fig. 6b) for 24 h did not significantly change the mRNA and protein expression of smad7, collagen 1, and α-SMA in TGFβ1-induced endometrial stromal cells after stimulation with VEGF165. However, qPCR and western blot analysis (Fig. 6c) of the cells treated with DAPT (30 μM) revealed that smad7 expression was significantly attenuated and that collagen 1 and α-SMA expression were increased after DAPT treatment, indicating that Notch signaling activation is the main pathway connecting VEGF165 and smad7 for alleviation of TGFβ1-induced α-SMA and collagen 1 expression.

### VEGF165 activates Notch signaling to inhibit TGFβ1-stimulated α-SMA and collagen 1 expression

**Fig. 7:**
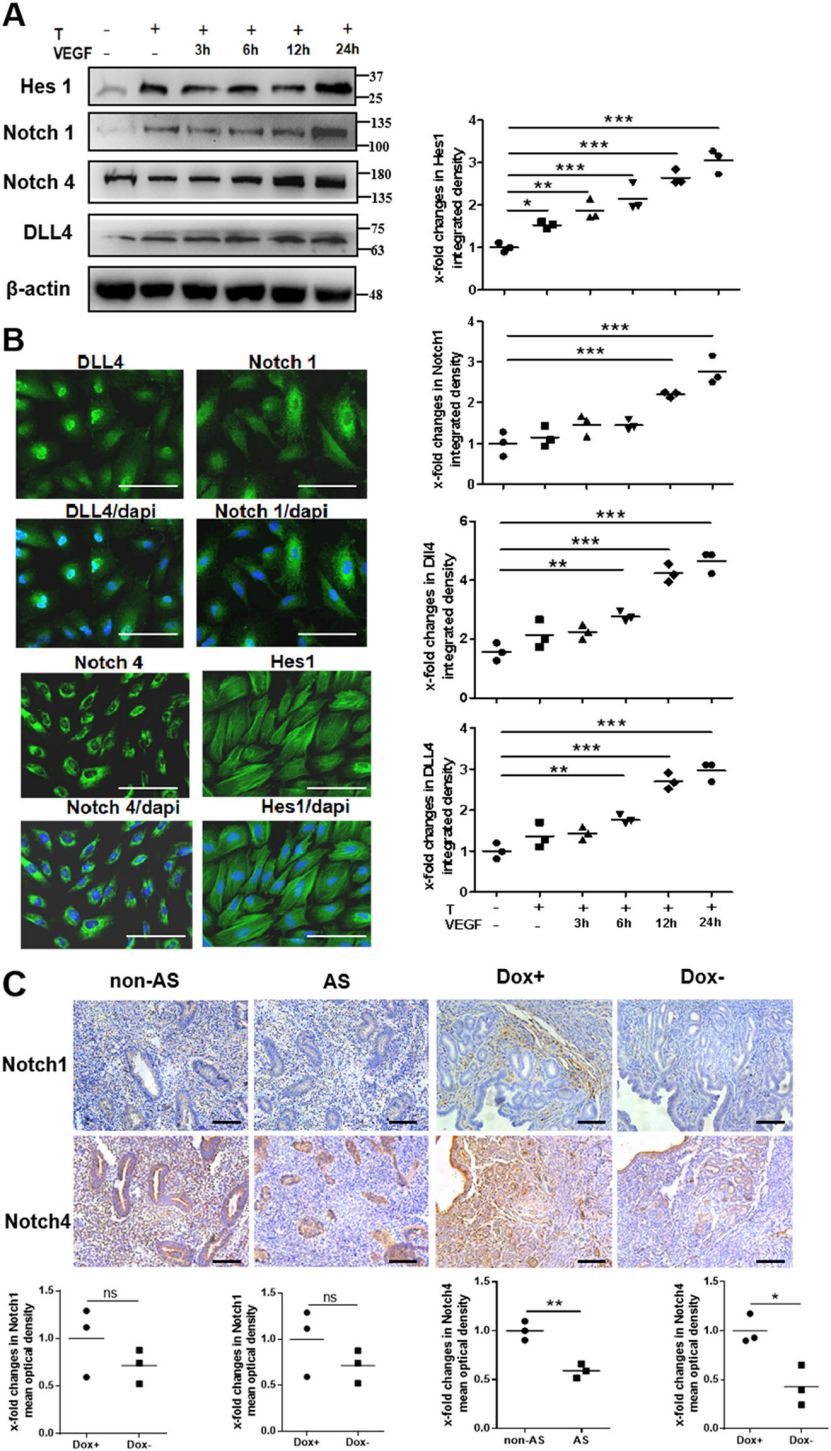
VEGF165 controls Notch signaling in endometrial stromal cells induced by TGFβ1. **a** The protein levels of DLL4, Notch1, Notch4, and Hes1 were examined by western blot analysis after ESCs were treated with 10 ng/mL TGFβ1 for 48 h and/or treated with 10 ng/mL VEGF165 for another 3, 6, 12, or 24 h. *N* = 3, ***P < 0.001, **P < 0.01, *P < 0.05. One-way ANOVA and SNK-q. **b** Representative images of DLL4, Notch1, Notch4, and Hes1 immunofluorescence staining in ESCs. Scale bar = 100 μm. *N* = 3, T: TGFβ1, VEGF: VEGF165. **c** Immunohistochemistry showed the expression patterns of Notch1 and Notch4 in human endometria at the late proliferative phase and in murine uteri in estrus. Scale bar = 100 μm. *N* = 3, **P < 0.01, *P < 0.05. Unpaired *t*-test

The mammalian family of Notch proteins consists of four receptors (Notch1–4) and a set of ligands comprising Jagged (Jag1 and Jag2) and Delta-like members (DLL1, DLL3, and DLL4)^23^. Binding of the ligands to a specific receptor activates Notch signaling, ultimately leading to transcriptional activation of downstream target genes, such as the members of the Hey and Hes families^25^. The mRNA and protein levels of DLL4, Notch1, and Notch4 in cultured endometrial stromal cells were increased in the group treated with TGFβ1 followed by VEGF165 compared to the control group and the group treated with TGFβ1 only (Fig. 6d, e). In addition, VEGF165 treatment could lead to increases in the levels of Hes1, Notch1, Notch4, and DLL4 in a time-dependent manner (Fig. 7a). The expression patterns of Hes1, Notch1, Notch4, and DLL4 in the endometrial stromal cells were confirmed by immunofluorescence (Fig. 7b).

### Notch4, not Notch1, mainly participates in the antifibrotic effect of VEGF165

The expression patterns of Notch1 and Notch4 in the endometria of AS patients at the late proliferative phase and in the uteri of Dox− mice at estrus were examined by immunohistochemistry (Fig. 7c). Notch1 was rarely expressed in the human endometrium or murine uterus, and there were no significant differences in Notch1 expression between the non-AS and AS groups or between the Dox+ and Dox− groups. In contrast, Notch4 was relatively highly expressed and was localized at the cytomembrane. The expression of Notch4 in the non-AS and Dox+ groups was significantly higher than that in the AS and Dox− groups, respectively. Notably, the differences in Notch4 mainly existed in the endometrial stroma (Fig. 7c).

**Fig. 8:**
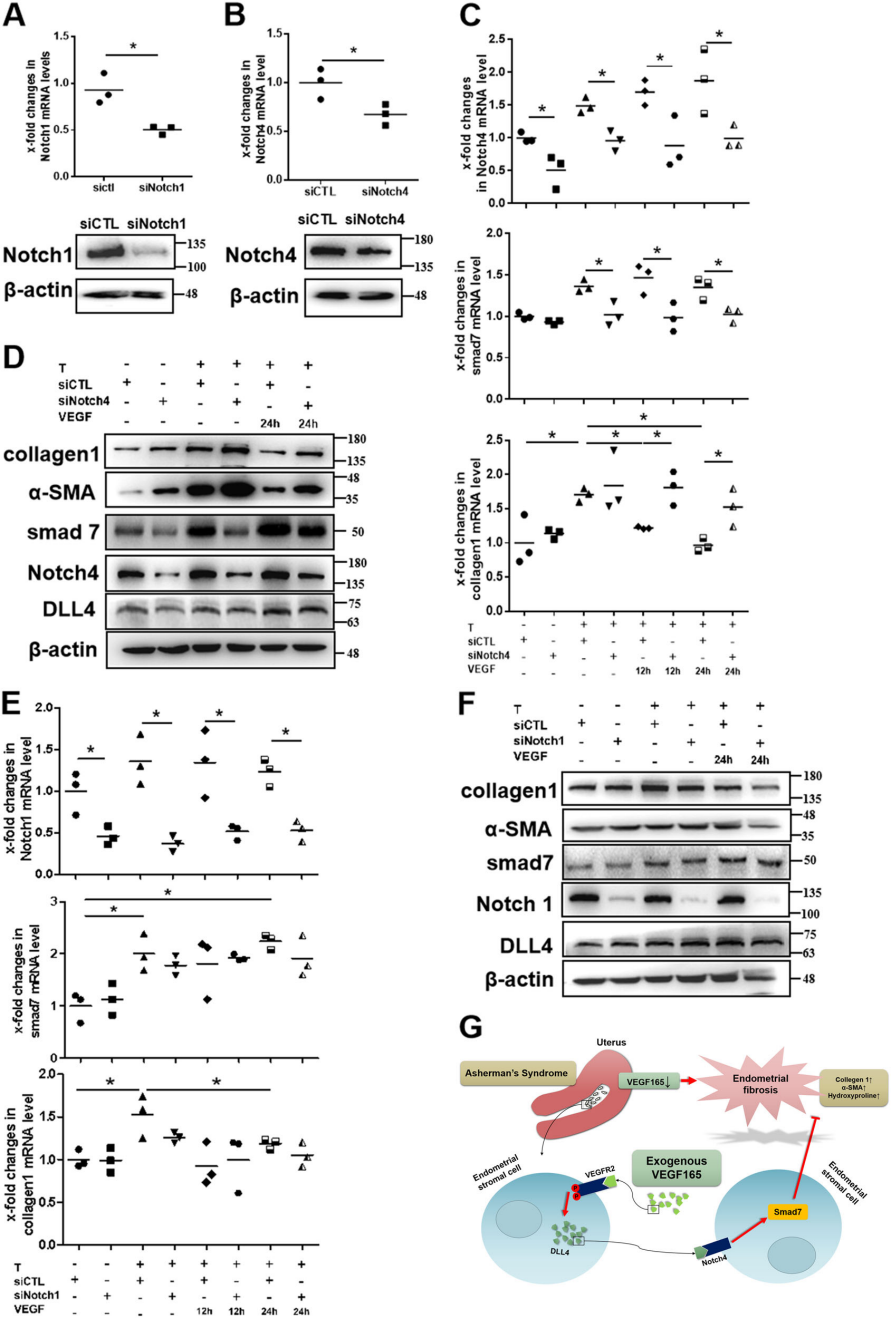
VEGFA-mediated promotion of smad7 expression mainly occurs through Dll4/Notch4 signaling. **a**, **b** The mRNA and protein levels of Notch1 and Notch4 in human endometrial stromal cells were decreased significantly at 24 h after transfection with siNotch1 or siNotch4, respectively. *N* = 3, *P < 0.05. Unpaired *t*-test. **c–f** Endometrial stromal cells stimulated with 10 ng/mL TGFβ1 for 48 h were transfected with siNotch1 and siNotch4 for 24 h and/or cocultured with 10 ng/mL VEGF165 for another 12 or 24 h. The mRNA and protein levels of Notch1, Notch4, smad7, α-SMA, and collagen 1 were examined. *N* = 3, *P < 0.05. Two-way ANOVA and SNK-q. T: TGFβ1, VEGF: VEGF165. **g** A model illustrating the role of VEGF165 in inhibiting endometrial fibrosis via DLL4/Notch4/Smad7 signaling. VEGF165 expression is decreased in the endometrium in Asherman’s syndrome, especially in stromal cells, which results in endometrial fibrosis, manifested as increased collagen 1 and α-SMA expression and hydroxyproline levels. Exogenous VEGF165 can reverse stromal cell differentiation into myofibroblasts and endometrial fibrosis by binding to VEGFR2 to induce DLL4/Notch4/Smad7 signaling

To further explore the role of Notch1 and Notch4 in TGFβ1-induced endometrial stromal cells treated with VEGF165, small interfering RNA-mediated knockdown of Notch1 (with siNotch1) and Notch4 (with siNotch4) was performed in stromal cells. As expected, both siNotch1 and siNotch4 efficiently reduced Notch1 and Notch4 expression, respectively, compared to the negative control, as shown by qPCR and western blot analysis (Fig. 8a, b). Analysis of the mRNA and protein levels of smad7 and collagen 1 showed that siNotch1 did not influence the antifibrotic effect of VEGF165, while siNotch4 significantly inhibited the decreased expression of fibrotic genes in stromal cells treated with VEGF165 (*P* < 0.05) (Fig. 8c–f), suggesting a main role of Notch4, but not Notch1, in the antifibrotic process of VEGF165.

## Discussion

In this study, decreased expression of VEGF165 was shown to cause the increased expression of α-SMA and collagen 1 and a phenotype of fibrosis in murine endometria. Treatment with exogenous VEGF165 in vitro inhibited TGFβ1-induced pro-fibrotic differentiation of stromal cells by promoting smad7 via DLL4/Notch4 signaling.

Studies have reported that VEGF165 not only maintains endothelial cell homeostasis but also plays an important role in the proliferation, differentiation, and function of nonendothelial cells^10,26,27^. Findings across multiple organ systems and model organisms have implicated VEGF165 in the control of regeneration and fibrosis. Stockmann et al.^11^ deleted the VEGF165 gene in myeloid cells and found that this deletion resulted in aggravated fibrotic tissue damage in a model of pulmonary fibrosis. Similar results were obtained by Yang et al.^12^, who used a murine model of liver fibrosis resolution and found that giving injections of VEGF-neutralizing antibody (mcr84) to the mice disrupted hepatic tissue repair and fibrosis resolution. Previously we reported that injection of a collagen-binding VEGFA, made by fusing a collagen-binding domain to the N-terminus of native VEGFA, into a rat scarred uterus model could promote regeneration of the endometrium and muscle cells, promote vascularization, and ultimately improve pregnancy outcomes^28^. In present study, we found that the expression of VEGF165 in the endometrium was significantly decreased and that the expression of VEGFR1 in the endometrial stroma was increased in AS patients. Repression of VEGF165 augmented collagen 1 and α-SMA expression and hydroxyproline content in human and murine endometrial stromas. Silencing VEGF165 aggravated the increased fibrotic gene expression, which could be hindered by restoration of VEGF165 protein in endometrial stromal cells. These findings indicated that VEGF165 reversed endometrial fibrosis mainly by inhibiting the pro-fibrotic differentiation of stromal cells.

Yang et al.^12^ attributed the antifibrotic effects of VEGFA to promotion of the production of the chemokine C-X-C-motif ligand 9 (CXCL9) and matrix metalloproteinase 13 by scar-associated macrophages, which resulted in extracellular matrix degradation. Herein, our data demonstrated that treatment with VEGF165 could significantly downregulate the increased collagen 1 and α-SMA in TGFβ1-stimulated stromal cells. Activation of the TGFβ1/Smad-dependent canonical pathway and a lack of negative feedback for this signaling upon injury are the main inducers of extracellular matrix (ECM) production and fibroblast-to-myofibroblast conversion^29^. Currently overwhelming evidence indicates that smad7 supplementation or induction is an effective method for treating fibrosis^30,31^. In our study, we showed that smad7 was reduced in the endometria of patients with AS and in the uteri of VEGF164-repressed mice, mainly in the endometrial stroma; we also demonstrated that VEGF165-deleted stromal cells had lower expression of smad7 than wild-type cells in vitro. Smad7 expression could dramatically increase in TGFβ1-induced endometrial stromal cells treated with VEGF165, indicating that VEGF165 and smad7 interact to counteract fibrosis. Furthermore, interference with smad7 in cells impaired the antifibrotic function of VEGF165, suggesting that smad7 is crucial in this function.

To gain molecular mechanistic insight regarding the enhancing effect of VEGF165 on smad7 expression to counteract fibrosis, we assessed Wnt/β-catenin, Hedgehog and Notch signaling to explore the underlying connection between VEGF165 and smad7, since these three pathways are known to play major roles in cell survival and differentiation^32–34^. Our data demonstrated that inhibition of Notch signaling, rather than Wnt/β-catenin or Hedgehog signaling, significantly attenuated the antifibrotic function of VEGF165, suggesting the key role of Notch signaling in the link between VEGF165 and smad7. These findings are consistent with those of cardiac studies demonstrating that Notch inhibition accelerates fibroblast-to-myofibroblast transformation and leads to the development of fibrosis^29,35,36^. Similar results were also obtained by Yang et al.^37^, who confirmed that myeloid-specific Notch activation ameliorates renal fibrosis, which is mediated by CCR2^+^ macrophage infiltration. In our studies, key components of the Notch pathway (DLL4, Notch1, and Notch4) were detected in endometrial stromal cells. However, Notch1 expression was very low in the late proliferative human endometrium and in the murine uterus during estrus. Previous studies have reported that VEGF165 signaling through VEGFR2 upregulates DLL4 expression in angiogenic vessels^25,38^. DLL4 is expressed in both epithelial and stromal cells of the endometrium and appears to be more efficient in stromal cells^39^. DLL4 can bind Notch1 and Notch4 in the endometrium^39,40^. Notch1 seems to be more involved in differentiation programming, while Notch4 seems to be more involved in controlling proliferation^41^. To further clarify the roles of Notch1 and Notch4 in the antifibrotic effect of VEGF165, we used siRNAs against Notch1 and Notch4 and found that only silencing Notch4 could significantly impair VEGF165-mediated induction of smad7 to downregulate fibrotic gene expression.

Experimental progress in identifying the roles of VEGF165 in mechanisms of fibrogenesis and fibrosis regression has been increasingly challenging due to the dependence of these roles on specific organs or microenvironments^11^. We developed a murine model of doxycycline-controlled VEGFA expression and repressed the level of VEGFA only after the mice matured; this model simulates the changes in VEGFA that occur in human diseases better than other models. Recent studies have reported that different subpopulations of fibroblasts exist whose proportions change during skin generation, wound healing, and fibrosis^42^. Thus, fibroblast diversity and the relationships between the subpopulations and VEGF165 expression in endometrial regeneration and fibrosis deserve further investigation. Additionally, whether upstream molecules and noncoding RNAs participate in regulating VEGF165 expression during the antifibrotic process needs to be investigated.

In summary, our findings highlight the importance of VEGF165 in inhibiting pro-fibrotic differentiation of stromal cells and show that DLL4/Notch4 signaling is required for VEGF165-induced smad7 expression to counteract endometrial stromal fibrosis (Fig. 8g). These findings are stepping stones for the development of therapeutic strategies to promote efficient endometrial repair and to treat fibrosis.

## Supplementary information

Supplementary Figure Legends

Figure S1

Figure S2

Figure S3

Figure S4

Figure S5

Figure S6

Figure S7

Table S1

Table S2
