# Supplementary material for: Vascular endothelial growth factor 165 inhibits pro-fibrotic differentiation of stromal cells via the DLL4/Notch4/smad7 pathway
Source: Cell Death Dis. 2019 Sep 12;10(9):681. doi: 10.1038/s41419-019-1928-z (PMC6742656; doi:10.1038/s41419-019-1928-z)
Supplement: Supplementary file 1 — Supplementary Figure Legends [file 41419_2019_1928_MOESM1_ESM.doc]

**Supplementary** **Figure Legends**

**Fig.S1** Negative control of immunohistochemistry for human endometrium (**a**) and murine uterus (**b**).

**Fig.S2** Paraffin-embedded human endometrial tissues were analyzed by immunohistochemistry for VEGFA, VEGFR1, VEGFR2, α-SMA, collagen 1, smad7, Notch1 or Notch4 and Masson trichrome-staining at the late proliferative phase. All images are magnified X400. Scale bar=100μm.

**Fig.S3** The exprssions of α-SMA in the human endometrium were examined by immunohistochemical staining, including six non-AS samples and six AS samples. α-SMA was mainly located in cytoplasm. (A) Normal endometrium had rare α-SMA expression or only around the vessels. (B) α-SMA increased dramatically in the endometrial stroma of AS patients and its signals were mainly around uterine glands. All images are magnified X200. Scale bar=100μm

**Fig.S4** Representative micrographs showed VEGFA, Ki67, active caspase, collagen 1, Smad7, Notch1 and Notch4 by immunohistochemistry and Masson trichrome-staining of murine uterus in estrus. All images are magnified X400. Scale bar=100μm.

**Fig.S5** Representative images from human endometiral stromal cells after being transfected by siControl (A) and siVEGF165(B) for 48h. Scale bar=100 μm.

**Fig.S6** Protein levels of collagen 1 and Smad7 were examined by western blot after endometrial stromal cells (ESCs) being treated with 10ng/mL VEGF165 (**a**) or TGFβ1 (**b**) for 3,6,12 or 24h. T: TGFβ1, VEGF: VEGF165.

**Fig. S7 Effects of different dosage of DAPT, Cyc and KYA1797K on Notch, Hedgehog and Wnt signaling respectively were examined by Western blot**. (**a**) ESCs which were induced by 10ng/mL TGFβ1 for 24h were treated with 0, 7.5, 15, 30, 45 or 60 μM DAPT for 24h, followed by 10ng/ml VEGF165 for 24h. (**b**) ESCs which were induced by 10ng/mL TGFβ1 for 24h were treated with 0, 2, 5, 10, 20 or 40 μM Cyc for 24h, followed by 10ng/ml VEGF165 for 24h. Cyc: cyclopamine. (**c**) ESCs which were induced by 10ng/mL TGFβ1 for 24h were treated with 0, 5, 10, 25, 50 or 75 μM KYA1797K for 24h, followed by 10ng/ml VEGF165 for 24h. Cyc: cyclopamine.
