# Supplementary figures and images for: Vascular endothelial growth factor 165 inhibits pro-fibrotic differentiation of stromal cells via the DLL4/Notch4/smad7 pathway
Source: Cell Death Dis. 2019 Sep 12;10(9):681. doi: 10.1038/s41419-019-1928-z (PMC6742656; doi:10.1038/s41419-019-1928-z)

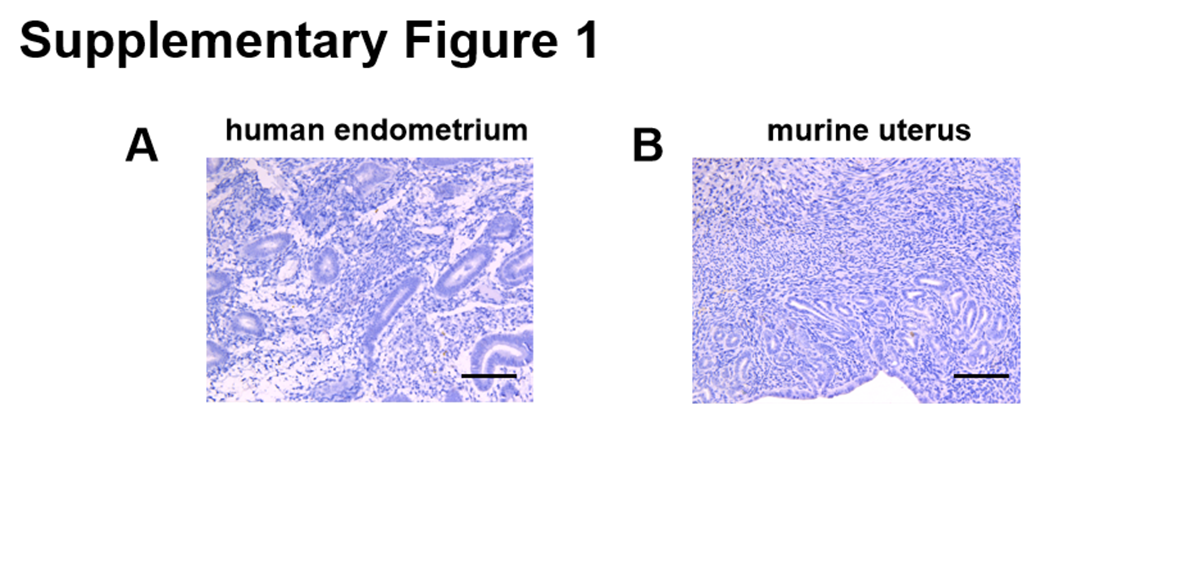

Supplement: Supplementary file 2 — Figure S1 [file 41419_2019_1928_MOESM2_ESM.tif]

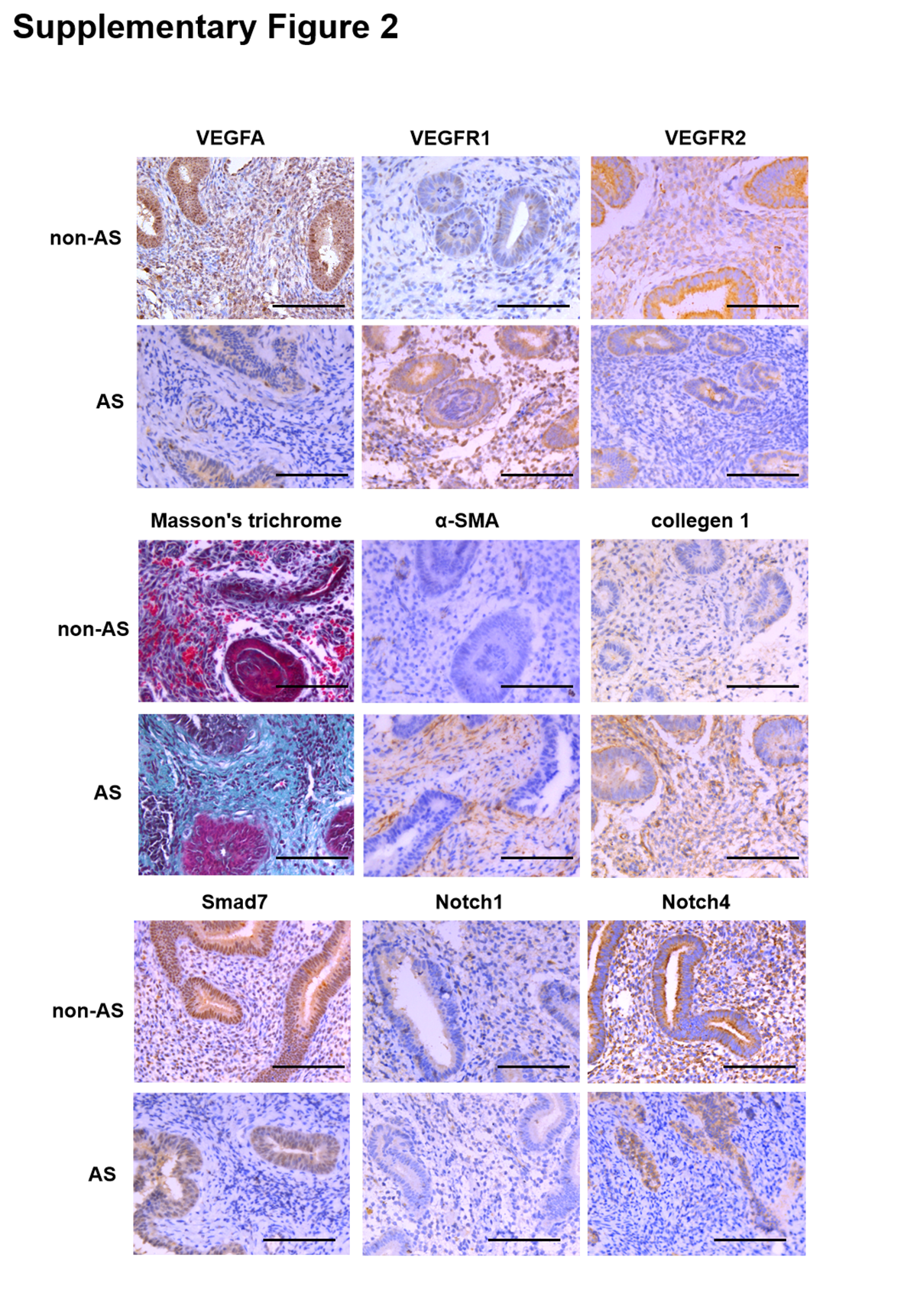

Supplement: Supplementary file 3 — Figure S2 [file 41419_2019_1928_MOESM3_ESM.tif]

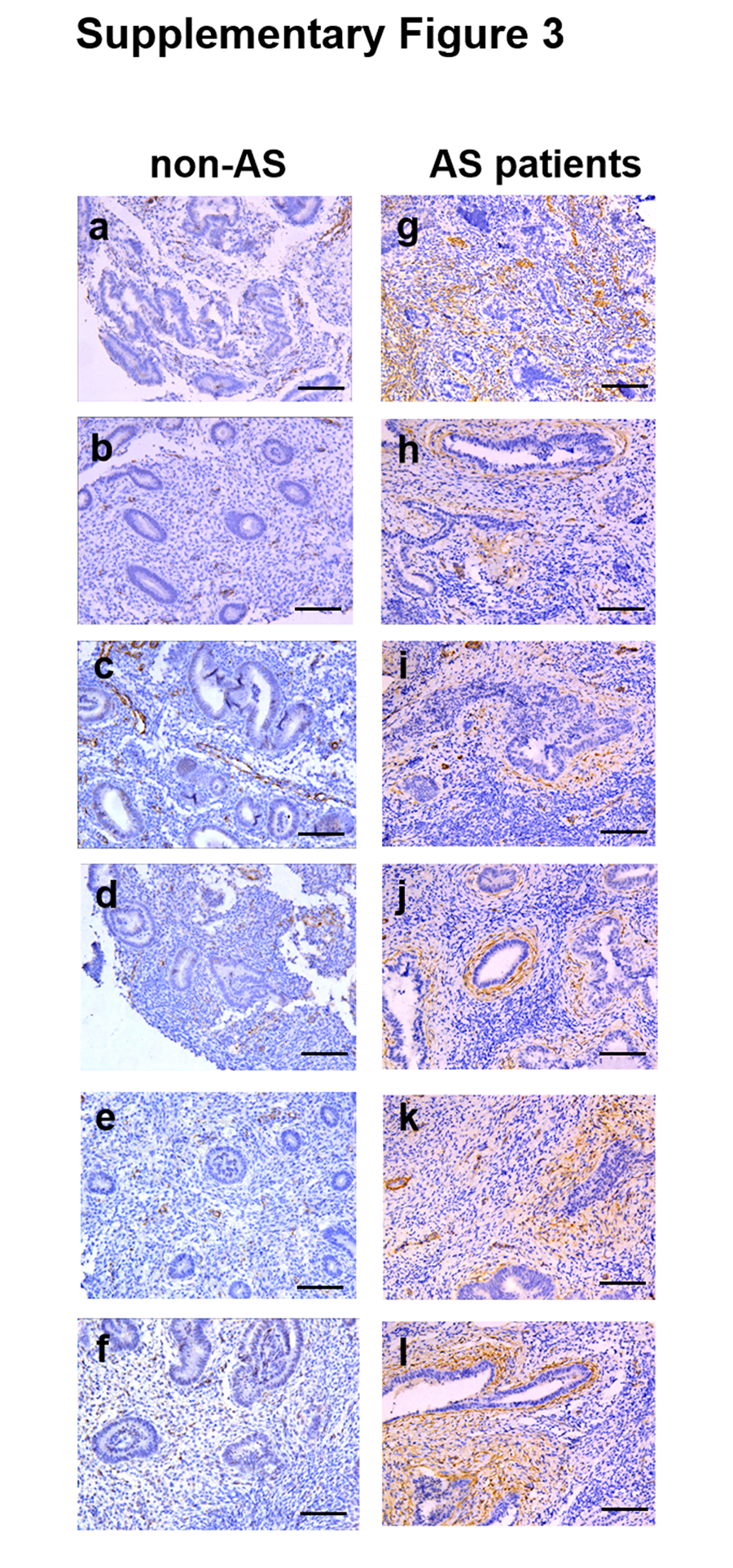

Supplement: Supplementary file 4 — Figure S3 [file 41419_2019_1928_MOESM4_ESM.tif]

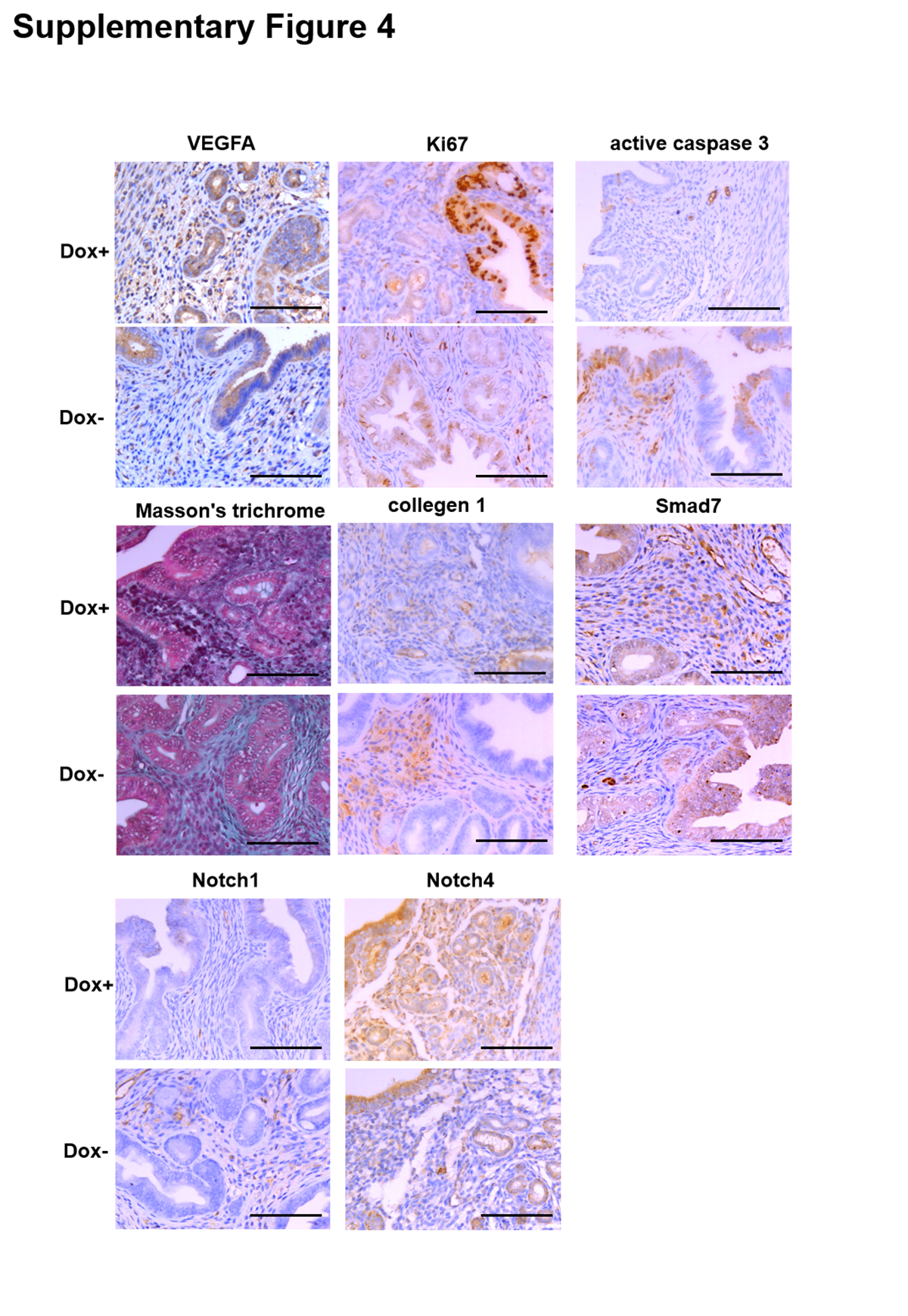

Supplement: Supplementary file 5 — Figure S4 [file 41419_2019_1928_MOESM5_ESM.tif]

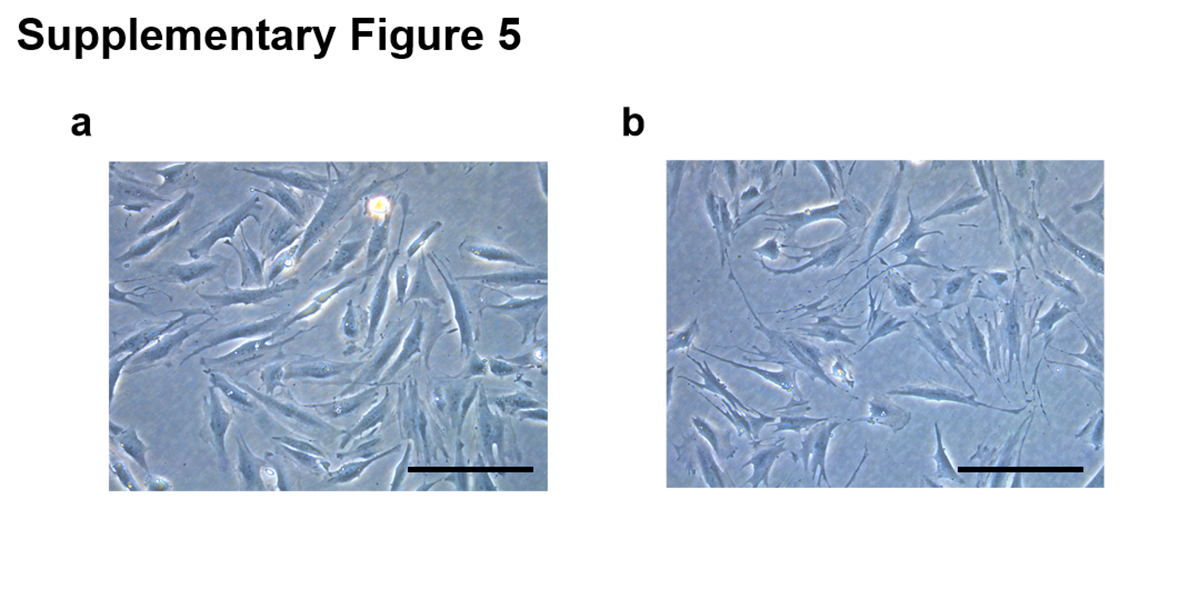

Supplement: Supplementary file 6 — Figure S5 [file 41419_2019_1928_MOESM6_ESM.tif]

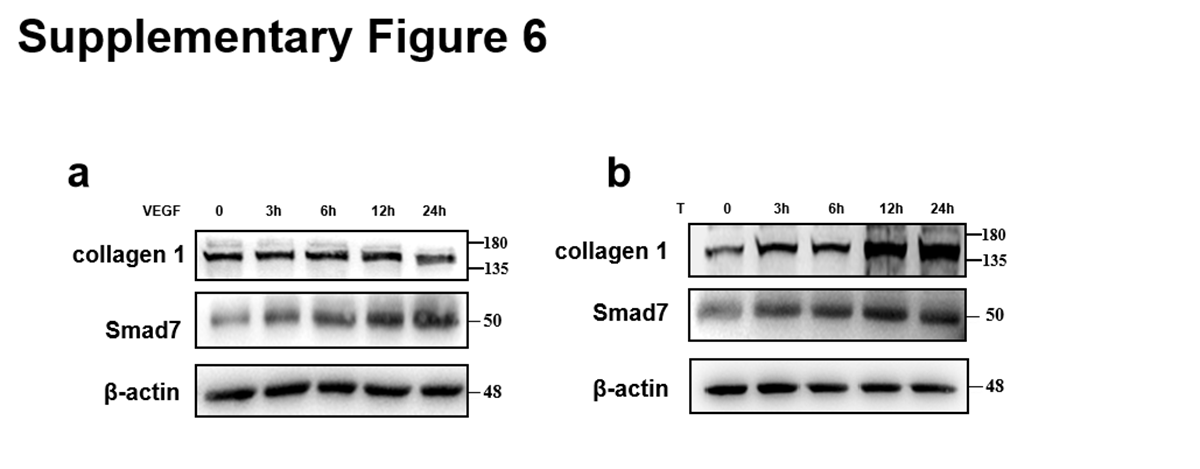

Supplement: Supplementary file 7 — Figure S6 [file 41419_2019_1928_MOESM7_ESM.tif]

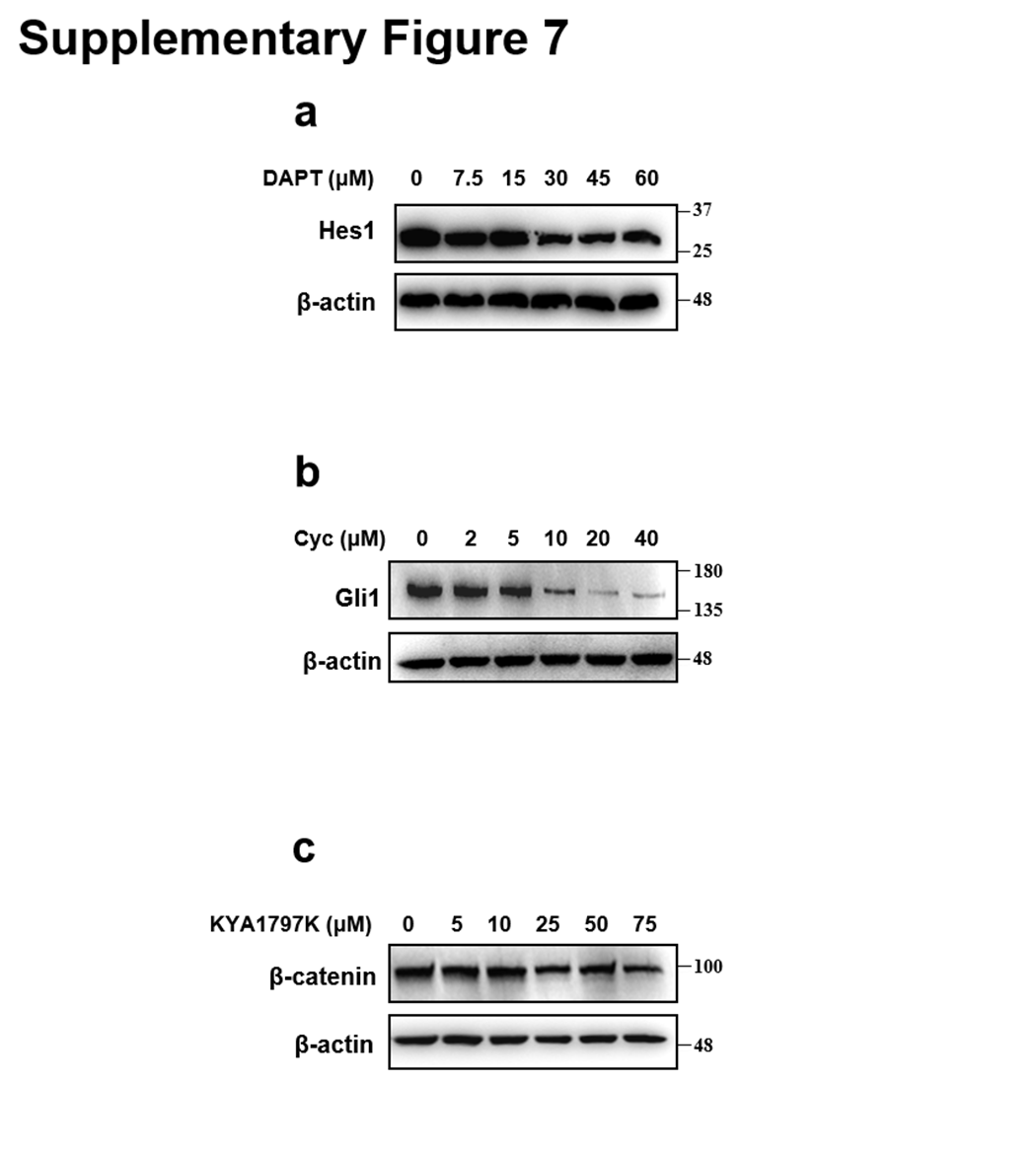

Supplement: Supplementary file 8 — Figure S7 [file 41419_2019_1928_MOESM8_ESM.tif]
